# Demographic Characteristics, Cigarette Smoking, and e-Cigarette Use Among US Adults

**DOI:** 10.1001/jamanetworkopen.2020.20694

**Published:** 2020-10-13

**Authors:** Margaret Mayer, Carolyn Reyes-Guzman, Rachel Grana, Kelvin Choi, Neal D. Freedman

**Affiliations:** 1 Tobacco Control Research Branch, Behavioral Research Program, Division of Cancer Control and Population Sciences, National Cancer Institute, National Institutes of Health, Bethesda, Maryland; 2 Division of Intramural Research, National Institute on Minority Health and Health Disparities, National Institutes of Health, Bethesda, Maryland; 3 Metabolic Epidemiology Branch, Epidemiology and Biostatistics Program, Division of Cancer Epidemiology and Genetics, National Cancer Institute, National Institutes of Health, Bethesda, Maryland

## Abstract

This cross-sectional study examines the demographic characteristics of adult cigarette smokers and e-cigarette users in the US.

## Introduction

Understanding how electronic cigarettes (e-cigarettes) are used by current, former, and never cigarette smokers may inform public health actions and tobacco regulations. Therefore, we examined the distribution of e-cigarette use, also called *vaping*, in the 2018-2019 Tobacco Use Supplement to the Current Population Survey, the largest nationally representative tobacco use survey of US adults.

## Methods

This cross-sectional study was determined to be exempt from review by an institutional review board at the National Institutes of Health because it was not human subjects research and used deidentified public use data. Informed consent was obtained prior to interviews by the US Census Bureau, which conducted the field work. In this study, adults aged 18 years and older were interviewed by phone (two-thirds of respondents) or in-home (one-third of respondents) once between July 2018 and May 2019 using probability-based multistage sampling.^1^ Among 137 471 self-respondents (self-response rate = 57.6%), we examined current e-cigarette use by demographic characteristics and cigarette smoking status. We also assessed whether former and current cigarette smokers who vape reported using e-cigarettes to help them quit smoking. This study followed the Strengthening the Reporting of Observational Studies in Epidemiology (STROBE) reporting guideline.

Weighted frequencies and proportions for statistical analysis were estimated with SAS-Callable SUDAAN (SAS version 9.4 [SAS Institute]; SUDAAN version 11.0.3 [RTI International]) using self-response survey weights. Data analysis was performed from October 2019 to July 2020.

## Results

The 2018-2019 Tobacco Use Supplement to the Current Population Survey was weighted to reflect the demographic characteristics of the US adult population. Our analytic sample included an unweighted total of 135 211 individuals with information on both cigarette and e-cigarette use (73 853 women [weighted percentage, 51.9%], weighted mean [SE] age 47.5 years [0.0]), of which 16 570 were current smokers (11.4% [95% CI, 11.2%-11.6%]), 29 189 were former smokers (18.2% [95% CI, 18.0%-18.5%]), and 90 906 were never cigarette smokers (70.3% [95% CI, 70.0%-70.7%]).

Overall, we estimate that more than 5.66 million adults in the US population reported current vaping (2.3% [95% CI, 2.2%-2.4%]). Among e-cigarette users, more than 2.21 million were current cigarette smokers (39.1% [95% CI, 36.8%-41.4%]), more than 2.14 million were former smokers (37.9% [95% CI: 35.6%-40.1%]), and more than 1.30 million were never smokers (23.1% [95% CI, 20.8%-25.4%]) (Table 1).

**Table 1.** Prevalence of Current Vaping by Cigarette Smoking Status, 2018-2019 Tobacco Use Supplement to the Current Population Survey^a^

Group	No. (weighted %)	Current vaping							
		All (N = 135 211)		Current cigarette smokers (n = 15 982)^b^		Former cigarette smokers (n = 28 890)^c^		Never cigarette smokers (n = 90 339)^d^	
		Unweighted No.	Weighted No (%) [95% CI]^e^	Unweighted No.	Weighted No. (%) [95% CI]^f^	Unweighted No.	Weighted, No. (%) [95% CI]^g^	Unweighted No.	Weighted No. (%) [95% CI]^h^	
Overall	135 211 (100.0)^i^	2747	5 666 729 (2.3) [2.2-2.4]	1158	2 214 741 (8.1) [7.6-8.7]	1142	2 145 059 (4.8) [4.5-5.1]	447	1 306 929 (0.8) [0.7-0.8]	
Age group, y										
18-24	7557 (11.9)	459	1 555 926 (5.3) [4.8-6.0]	127	392 325 (18.4) [15.0-22.3]	108	334 704 (26.8) [21.5-32.8]	224	828 896 (3.2) [2.7-3.8]	
25-34	21 248 (17.9)	753	1 566 900 (3.6) [3.3-3.9]	286	628 406 (11.9) [10.6-13.4]	330	626 856 (13.5) [11.8-15.4]	137	311 637 (0.9) [0.8-1.1]	
35-44	22 131 (16.3)	518	925 398 (2.3) [2.1-2.6]	228	390 590 (7.7) [6.6-8.9]	251	450 511 (7.2) [6.1-8.4]	39	84 297 (0.3) [0.2-0.4]	
45-54	20 632 (16.3)	403	693 564 (1.7) [1.5-2.0]	209	349 453 (6.6) [5.6-7.7]	168	294 972 (4.6) [3.8-5.5]	26	49 139 (0.2) [0.1-0.3]	
55-64	25 563 (16.8)	385	61 293 (1.5) [1.3-1.7]	202	316 195 (5.3) [4.4-6.3]	169	270 736 (2.9) [2.4-3.5]	14	26 001 (0.1) [0.1-0.2]	
≥65	38 080 (20.7)	229	312 010 (0.6) [0.5-0.7]	106	137 772 (3.9) [3.1-4.8]	116	167 279 (1.0) [0.8-1.2]	7	6959 (0.0) [0.0-0.1]	
Sex										
Male	61 358 (48.1)	1525	3 344 129 (2.8) [2.7-3.0]	613	1 274 449 (8.6) [7.8-9.4]	650	1 285 214 (5.1) [4.7-5.6]	262	784 467 (1.0) [0.9-1.2]	
Female	73 853 (51.9)	1222	2 322 600 (1.8) [1.7-2.0]	545	940 292 (7.5) [6.9-8.2]	492	859 845 (4.4) [4.0-4.8]	185	522 462 (0.5) [0.5-0.7]	
Race/ethnicity^j^										
Non-Hispanic White	98 689 (63.1)	2255	4 328 610 (2.8) [2.6-2.9]	952	1 679 627 (8.8) [8.2-9.5]	982	1 748 799 (5.0) [4.7-5.4]	321	900 183 (0.9) [0.8-1.0]	
Non-Hispanic Black	12 799 (11.8)	112	316 728 (1.1) [0.9-1.3]	51	135 253 (3.8) [2.8-5.2]	35	93 070 (2.8) [1.9-4.2]	26	88 405 (0.4) [0.3-0.6]	
Hispanic	14 671 (16.5)	206	613 399 (1.5) [1.3-1.8]	82	242 237 (8.1) [6.3-10.5]	61	155 513 (3.5) [2.6-4.8]	63	215 650 (0.6) [0.5-0.8]	
American Indian/Alaskan Native	1222 (0.7)	39	77 288 (4.2) [2.8-6.4]	17	28 305 (7.6) [3.9-14.2]	14	36 534 (10.5) [5.6-18.8]	8	12 448 (1.1) [0.5-2.5]	
Asian	5773 (6.1)	66	149 501 (1.0) [0.7-1.4]	26	58 368 (8.2) [5.2-12.6]	29	67 503 (5.2) [3.5-7.7]	11	23 630 (0.2) [0.1-0.4]	
Multiracial	1672 (1.5)	56	163 058 (4.5) [3.3-6.2]	22	63 600 (10.4) [6.3-16.9]	17	35 566 (5.3) [3.1-8.8]	17	63 893 (2.8) [1.6-4.7]	
Education										
Less than high school	11 360 (9.5)	227	510 130 (2.2) [1.8-2.6]	123	237 971 (5.7) [4.6-7.1]	72	132 601 (3.6) [2.6-4.9]	32	139 559 (0.9) [0.6-1.4]	
High school degree	35 897 (26.4)	922	1 919 182 (3.0) [2.7-3.2]	425	806 247 (7.6) [6.8-8.5]	371	729 222 (5.7) [5.1-6.4]	126	383 713 (0.9) [0.7-1.1]	
Some college	38 716 (28.7)	1065	2 159 209 (3.1) [2.8-3.3]	428	802 226 (9.2) [8.2-10.4]	453	815 503 (5.7) [5.1-6.4]	184	541 480 (1.1) [1.0-1.3]	
College degree	49 238 (35.4)	533	1 078 208 (1.2) [1.1-1.4]	182	368 297 (9.6) [8.1-11.4]	246	467 733 (3.3) [2.9-3.8]	105	242 177 (0.4) [0.3-0.4]	

^a^ Current vaping was defined as ever use of an e-cigarette and now vaping every day or some days. Current smokers had smoked 100 lifetime cigarettes and now smoked every day or some days; former smokers had smoked 100 lifetime cigarettes and now smoked not at all; never smokers had not smoked 100 lifetime cigarettes.

^b^ Weighted proportion of current vapers who are current cigarette smokers is 39.1% (95% CI, 36.8%-41.4%).

^c^ Weighted proportion of current vapers who are former cigarette smokers is 37.9% (95% CI, 35.6%-40.1%).

^d^ Weighted proportion of current vapers who are never cigarette smokers is 23.1% (95% CI, 20.8%-25.4%).

^e^ Weighted percentages represent the prevalence of current vaping overall and in each demographic subgroup.

^f^ Weighted percentages represent the prevalence of current vaping among current cigarette smokers overall and among demographic subgroups of current cigarette smokers.

^g^ Weighted percentages represent the prevalence of current vaping among former cigarette smokers overall and among demographic subgroups of former cigarette smokers.

^h^ Weighted percentages represent the prevalence of current vaping among never cigarette smokers overall and among demographic subgroups of never cigarette smokers.

^i^ Excludes 2260 individuals with missing information for cigarette use or e-cigarette use.

^j^ Hawaiian/Pacific Islander results not presented because of small cell sizes.

The prevalence of vaping was higher among men (2.8%; 95% CI, 2.7%-3.0%) and among non-Hispanic White (2.8% [95% CI, 2.6%-2.9%]), American Indian/Alaskan Native (4.2% [95% CI, 2.8%-6.4%]), and multiracial (4.5% [95% CI, 3.3%-6.2%]) individuals. There was higher prevalence with increasing education level (less than high school: 2.2% [95% CI, 1.8%-2.6%]; high school degree: 3.0% [95% CI, 2.7%-3.2%]; and some college: 3.1% [95% CI, 2.8%-3.3%]), except for individuals with a college degree (1.2% [95% CI, 1.1%-1.4%]), who had the lowest prevalence. Across all categories of sex, race/ethnicity, and education, the majority of vapers were current or former smokers. There were, however, differences by age. Among never smokers who vaped, 63.4% (95% CI, 58.2%-68.7%) were between 18 and 24 years old, and 23.8% (95% CI, 19.6%-28.1%) were between 25 and 34 years old. In contrast, e-cigarette users who were current or former smokers tended to be older (Table 1).

Among current dual users of cigarettes and e-cigarettes, 69.3% (95% CI, 65.7%-72.7%) reported using e-cigarettes to try to quit smoking. However, among former smokers who currently vape, 80.7% (95% CI, 77.4%-83.5%) reported that they had used e-cigarettes to help them quit smoking (Table 2).

**Table 2.** Self-reported Use of e-Cigarettes to Quit Smoking Among Current and Former Cigarette Smokers Who Currently Vape, 2018-2019 Tobacco Use Supplement to the Current Population Survey

Group	Current dual users of cigarettes and e-cigarettes			Former smokers who currently use e-cigarettes		
	Overall No.	Report using e-cigarettes to help quit cigarette smoking, weighted % (95% CI)		Overall No.	Report that they used e-cigarettes to help quit cigarette smoking, weighted % (95% CI)	
		No	Yes		No	Yes
Overall	1151	30.7 (27.3-34.3)	69.3 (65.7-72.7)	1136	19.3 (16.5-22.6)	80.7 (77.4-83.5)
Age group, y						
18-24	126	47.2 (36.4-58.3)	52.8 (41.7-63.6)	108	19.0 (11.4-30.1)	81.0 (69.9-88.6)
25-34	285	24.8 (19.5-31.1)	75.2 (68.9-80.5)	329	18.5 (14.1-24.0)	81.5 (76.0-85.9)
35-44	226	29.6 (23.4-36.5)	70.4 (63.5-76.6)	250	17.2 (12.6-23.0)	82.8 (77.0-87.4)
45-54	208	22.3 (16.1-30.0)	77.7 (70.0-83.9)	167	23.4 (16.8-31.6)	76.6 (68.4-83.2)
55-64	202	31.5 (23.6-40.6)	68.5 (59.4-76.4)	168	18.0 (11.9-26.4)	82.0 (73.6-88.1)
≥65	104	33.5 (24.3-44.1)	66.5 (55.8-75.7)	114	23.8 (15.1-35.5)	76.2 (64.5-84.9)
Sex						
Male	608	33.9 (29.2-38.9)	66.1 (61.1-70.8)	648	19.9 (16.4-24.1)	80.1 (75.9-83.6)
Female	543	26.4 (22.4-30.9)	73.6 (69.1-77.6)	488	18.4 (14.3-23.4)	81.6 (76.6-85.7)
Race/ethnicity^a^						
Non-Hispanic White	948	28.6 (24.8-32.7)	71.4 (67.3-75.2)	977	18.2 (15.3-21.6)	81.8 (78.4-84.7)
Non-Hispanic Black	51	39.0 (25.4-54.5)	61.0 (45.5-74.6)	35	28.4 (14.8-47.6)	71.6 (52.4-85.2)
Hispanic	80	38.8 (26.7-52.3)	61.2 (47.7-73.3)	61	21.9 (12.2-36.2)	78.1 (63.8-87.8)
American Indian–Alaskan Native	17	65.4 (40.7-83.9)	34.6 (16.1-59.3)	14	4.8 (0.6-30.1)	95.2 (69.9-99.4)
Asian	26	19.1 (6.9-42.9)	80.9 (57.1-93.1)	28	34.8 (16.8-58.7)	65.2 (41.3-83.2)
Multiracial	21	34.4 (14.8-61.3)	65.6 (38.7-85.2)	17	30.8 (10.3-63.3)	69.2 (36.7-89.7)
Education						
Less than high school	123	43.7 (32.6-55.5)	56.3 (44.5-67.4)	72	25.0 (13.4-41.8)	75.0 (58.2-86.6)
High school degree	423	28.4 (23.3-34.2)	71.6 (65.8-76.7)	370	18.6 (14.4-23.8)	81.4 (76.2-85.6)
Some college	423	27.4 (22.2-33.2)	72.6 (66.8-77.8)	450	18.7 (14.3-24.1)	81.3 (75.9-85.7)
College degree	182	34.4 (26.7-43.1)	65.6 (56.9-73.3)	244	19.9 (15.0-26.1)	80.1 (73.9-85.0)

^a^ Hawaiian/Pacific Islander results are not presented because of small cell sizes.

## Discussion

We estimate a vaping prevalence of 2.3% among US adults. An estimated 39.1% of e-cigarette users were current smokers and 37.9% were former smokers. A majority of both groups reported using or having used e-cigarettes to help them quit cigarette smoking. Whether vaping actually helps smokers quit is unclear, however, and cannot be evaluated using cross-sectional data. Longitudinal studies and trials are needed to answer this important question and to determine the long-term health effects of dual use, which was the most common pattern of use reported. The large number of former smokers who currently vape is additionally worrisome, given recent reports that former smokers who vape are more likely to experience a smoking relapse.^2^

Additionally, 23.1% of e-cigarette users reported never smoking, and most were younger than 35 years. In addition to the potential health effects of vaping,^3^ the young age of many users is concerning, particularly as nicotine is highly addictive and can negatively affect brain development, which continues until age 25 years.^4^

Limitations of this study include a cross-sectional design and self-reported data. Strengths of these analyses include recency of the data and a large nationally representative sample, which makes the findings generalizable.

e-Cigarettes are now used by large numbers of never, former, and current cigarette smokers in the US population. Given the rapidly changing e-cigarette marketplace and recent increases in the prevalence of use among youth^5^ and young adults,^6^ continued surveillance is needed.
